# Clinical presentation and prognostic factors of eyelid sebaceous carcinoma

**DOI:** 10.1007/s10384-025-01187-2

**Published:** 2025-05-25

**Authors:** Yukiko Aihara, Masachika Ikegami, Yoshiyuki Suehara, Takane Watanabe, Seiichi Yoshimoto, Taisuke Mori, Hiroyuki Mano, Shinji Kohsaka, Shigenobu Suzuki

**Affiliations:** 1 Department of Ophthalmic Oncology, National Cancer Center Hospital, 5-1-1 Tsukiji, Chuo-ku, Tokyo, 104-0045 Japan; 2 Department of Ophthalmology, University of Tokyo, 7-3-1 Hongo, Bunkyo-ku, Tokyo, 113-8655 Japan; 3 Division of Cellular Signaling, National Cancer Center Research Institute, 5-1-1 Tsukiji, Chuo-ku, Tokyo, 104-0045 Japan; 4 Department of Head and Neck Surgery, National Cancer Center Hospital, 5-1-1 Tsukiji, Chuo-ku, Tokyo, 104-0045 Japan; 5 Department of Diagnostic Pathology, National Cancer Center Hospital, Tokyo, 5-1-1 Tsukiji, Chuo-ku, Tokyo, 104-0045 Japan

**Keywords:** Sebaceous carcinoma, Eyelid, Prognosis, Prognostic factor

## Abstract

**Purpose:**

To clarify the demographics, clinical features, outcomes, and prognostic factors of Japanese patients with eyelid sebaceous carcinoma.

**Study design:**

Retrospective study.

**Methods:**

Fifty-two patients with eyelid sebaceous carcinoma diagnosed histopathologically at the National Cancer Center Hospital in Japan between 2013 and 2023 were reviewed and their outcomes were examined.

**Results:**

There were 21 men and 31 women. The median age at diagnosis was 73 (range 39–95) years old. The tumor was located in the upper eyelid in 33 patients (63%), in the lower eyelid in 16 patients (31%), and in both eyelids in 3 patients (6%). The initial curative treatment included surgical resection with or without eyelid reconstruction in 41 cases (79%), orbital exenteration in 4 cases (8%), and radiation therapy in 7 cases (13%). Two- and five-year adjusted survival rates were 97.4% and 93.7%, respectively. Local recurrence occurred in 15 cases (29%). Lymph node metastasis was confirmed in 13 cases (27%), distant metastasis in 5 cases (10%), and 3 patients (6%) died of lung metastasis. T2b or more advanced T stage was associated with a risk of shorter overall survival (HR = 5.3, 95% CI = 1.2–23, *p* = 0.024) compared with T2a or earlier stage, as well as lymph node metastasis (HR = 82, 95% CI = 2.6–2.6e+03, *p* = 0.013). A positive surgical margin increased the risk of local recurrence (HR = 37, 95% CI = 5.9- 235, *p* = 0.00012).

**Conclusion:**

Proper diagnosis and margin-free resection are necessary before eyelid sebaceous carcinoma can further develop.

## Introduction

Sebaceous carcinoma (SC) is a rare and potentially aggressive malignancy primarily located in the head and neck. It originates from the sebaceous glands in the skin or Meibomian and Zeis glands in the eyelid. It often develops in the upper eyelid where Meibomian glands are abundant. There are racial differences in the prevalence of SC. It accounts for 0.5%–14% of all malignant eyelid neoplasms including basal cell carcinoma and squamous cell carcinoma in Western countries [1–3]; however, it is more frequent in Asian countries. For example, it accounts for 8% of malignant eyelid neoplasms in Taiwan [4], 30% in the Philippines [5], 31.7% in China [6], 28.9%–54% in Japan [7, 8], and 32.6%–55.7% in India [9–12]. Muir-Torre syndrome, a rare autosomal dominant disorder, causes cutaneous lesions including sebaceous neoplasms and visceral malignancies (primarily colorectal carcinomas). The gold standard treatment for resectable SC is surgical resection, whereas a basis for chemotherapeutic drug regimens has not been established for advanced SC. Radiation therapy is administered to some patients instead of surgery because of general conditions or cosmetic reasons [13]. If SC cannot be completely removed by surgery, radiation therapy may be administered in addition to surgery. Although small SC lesions that are resectable with negative margins are known to have a good prognosis, large SC lesions that are not completely resectable are potentially life-threatening because of recurrence and metastasis. There are some reports of Japanese eyelid SC; however, their prognostic factors have not been identified. We examined the clinical presentation and outcomes of Japanese eyelid SC patients to identify prognostic factors.

## Materials and methods

### Study design and ethics

This study was a retrospective chart review. The protocol for this retrospective observational study was approved by the Institutional Review Board of the National Cancer Center Japan (approval number: 2019-158). This study was conducted at the National Cancer Center Japan in accordance with the Declaration of Helsinki. All patients were treated with individual consent.

### Data collection

Medical records for 52 patients who had been histopathologically diagnosed with eyelid SC between 2013 and 2023 at the National Cancer Center Hospital in Japan were reviewed. Clinical information including sex, age, tumor location, type (nodular or diffuse), initial complaint, tunor nodes and metastases (TNM) classification [the eighth edition of American Joint Committee on Cancer (AJCC)], treatment information, presence/absence of pagetoid spread (intraepithelial invasion), invasion of adjacent tissue such as vessels, lymphatic vessels, muscles, nerves, and adnexa and surgical margin was collected. We also collected data on local recurrence, lymph node metastasis, distant metastasis, and prognosis. For T1 tumors with a negative surgical margin, we asked the doctor of the referral hospital to conduct a follow up. For T1 tumors with a positive surgical margin or for tumors of T2 grade or worse, we followed up at 1, 3, 6, 9, and 12 months after the surgery and every 6 months after 1 year. Because, in most cases lymph node metastasis occurs before distant organ metastasis, we checked the regional lymph node by palpation at every visit. Whenever lymph node metastasis was suspected, we performed further examinations such as cervical ultrasound scan and contrast-enhanced computed tomography to check for lymph node and distant metastasis as needed. Once lymph node metastasis was confirmed, we consulted head and neck surgeons about surgical intervention including neck dissection. After the surgery, computed tomography from the neck to the pelvis was performed every six months.

### Statistical analysis

Overall survival, recurrence-free survival, lymph node metastasis-free survival, and distant metastasis-free survival were evaluated from the initial curative treatment. Lymph node metastasis-free survival was evaluated only for N0 patients. Survival analysis was done using the Kaplan–Meier estimator and log-rank test, and a hazard ratio calculation was conducted with survival package version 3.6–4 for R. All statistical analyses were performed using R, version 4.3.2. Two-tailed p < 0.05 and a 95% confidence interval of the difference not containing zero were considered statistically significant.

## Results

### Patient characteristics

The study cohort consisted of 52 patients with eyelid SC histopathologically diagnosed and treated between 2013 and 2023 at the National Cancer Center Hospital in Japan. The demographic and clinical data of these patients are listed in Table 1. There were 21 men and 31 women, with the median age at the time of histopathological diagnosis of 73 (range 39–95) years old. All patients had unilateral eyelid SC: left eyelid in 26 patients (50%) and right eyelid in 26 patients (50%). The tumor was located in the upper eyelid in 33 patients (63%), in the lower eyelid in 16 patients (31%), and in both eyelids in 3 patients (6%). The tumor was present in the medial position in 23 patients (44%), in the middle position in 20 patients (38%), and in the lateral position in 9 patients (17%). The nodular type was in 48 patients (92%), and the diffuse type was in four patients (8%). The most frequent initial complaint was tumor mass (79%).

**Table 1:** Clinical data of eyelid sebaceous carcinoma.

Background data		Total (N = 52)
Sex	Male	21 (40%)
	Female	31 (60%)
Age	Median (range)	73 (39-95)
Laterality (left or right)	Left	26 (50%)
	Right	26 (50%)
Site (upper or lower)	Upper	33 (63%)
	Lower	16 (31%)
	Both	3 (6%)
Location (medial, middle or lateral)	Medial	23 (44%)
	Middle	20 (38%)
	Lateral	9 (17%)
Type (nodular or diffuse)	Nodular	48 (92%)
	Diffuse	4 (8%)
Initial complaint	Tumor mass	41 (79%)
	Eyelid swelling	5 (10%)
	Others	6 (12%)
TNM T category	T1a	21 (40%)
	T1b	1 (2%)
	T2a	10 (19%)
	T2b	7 (13%)
	T3a	1 (2%)
	T3b	2 (4%)
	T3c	3 (6%)
	T4a	7 (13%)
TNM N category	N0	47 (90%)
	N1	5 (10%)
TNM M category	M0	52 (100%)
Stage	IA	22 (42%)
	IB	10 (19%)
	IIA	10 (19%)
	IIB	5 (10%)
	IIIA	5 (10%)
Initial curative treatment	Surgery	45 (87%)
	Excision	32 (62%)
	Excision with reconstruction	9 (17%)
	Exenteration	4 (8%)
	Radiation	7 (13%)
Hospital of initial curative treatment	Our hospital	39 (75%)
	Other hospitals	13 (25%)
Surgical margin	Negative	36/45 (80%)
	Positive	9/45 (20%)
Pathological findings ^1)^	Pagetoid spread	6 (12%)
	Vascular invasion	6 (12%)
	Lymphatic invasion	2 (4%)
Prognosis ^1)^	Local recurrence	15 (29%)
	Metastasis	
	Regional lymph nodes	13 (25%)
	Distant organs	5 (10%)
	Tumor related death	3 (6%)

1) These contains duplicates, respectively.

According to the TNM criteria classification of the AJCC Cancer Staging Manual eighth edition, T1a was most frequent (21/52 patients, 40%). Lymph node metastasis was identified in five patients (10%) at the time of histopathological diagnosis of eyelid SC (N1). All lymph node metastases were in the ipsilateral parotid gland or the cervical lymph nodes. No distant metastases were observed at the time of the initial histopathological diagnosis (M0). Clinically, sebaceous neoplasms accompanied by visceral malignancies suggested the possibility of Muir-Torre syndrome. Immunostaining of mismatch repair genes such as *MLH1*, *MSH2*, *MSH6*, and *PMS2* was performed in four cases suspected Muir-Torre syndrome based on the medical history, but all immunostaining was positive. Therefore, there were no cases diagnosed with Muir-Torre syndrome in our cohort. Thirty-nine patients (75%) underwent the initial curative treatment at our hospital. Thirteen patients (25%) underwent the initial curative treatment in other hospitals and were transferred to our hospital because of recurrence or metastasis. A histopathological assessment revealed pagetoid spread in 6 patients (12%), vascular invasion in 6 patients (12%), and lymphatic invasion in 2 patients (4%).

### Treatment

Forty-five patients (87%) underwent surgical resection as the initial curative treatment, and seven patients (13%) received radiation therapy after incisional biopsy. Among those treated surgically, negative margins were achieved in 36 patients (80%), and positive margins in nine patients (20%). Among patients with positive margins, if the patient opted for follow-up, the tumor was monitored and resected if clinical recurrence occurred; if the patient opted for radiation therapy, the patient was treated with postoperative radiation therapy. In this study, five patients opted for follow-up and four for postoperative radiation therapy. The T1 patients did not require complex reconstruction (21/21, 100%), however, some of T2 patients (6/14, 43%) and most of T3 patients (2/3, 67%) needed complex reconstruction. Orbital exenteration was performed only for T4 patients (4/6, 67%). For large SCs that did not invade beyond the periosteum by an imaging test (T4a) without distant metastasis, we proposed orbital exenteration in the hope of local control. In our cohort, all T4 patients were classified as T4a. In our hospital, whenever we treat T4b patients with parenchymal invasion of the bone, we perform enlarged surgery such as an expanded total maxillectomy because T4b tumors cannot be cured by orbital exenteration. For the four cases with ipsilateral parotid or cervical lymph node metastasis at the initial diagnosis (N1), superficial parotidectomy with neck dissection was performed in addition to resection of the eyelid tumor. Postoperative radiation therapy to the neck including the parotid region was administered in three of these patients, because either extranodal extensions were observed out of the lymph node structure or four or more lymph node metastases were detected. In our cohort, all patients were M0. In cases with distant metastasis at the initial diagnosis (M1), we do not perform a large surgery because it will not result in a cure even if orbital tumors can be completely removed locally. Topical mitomycin C therapy was not performed in our hospital, and only two patients had already received it at the referring hospital. In the four cases of diffuse type, one T2b and one T3c patients underwent tumor resection with complex reconstruction, one T4a patient underwent orbital exenteration, and one T3b patient underwent radiation therapy.

### Prognosis and local recurrence after the initial treatment

**Fig. 1.**
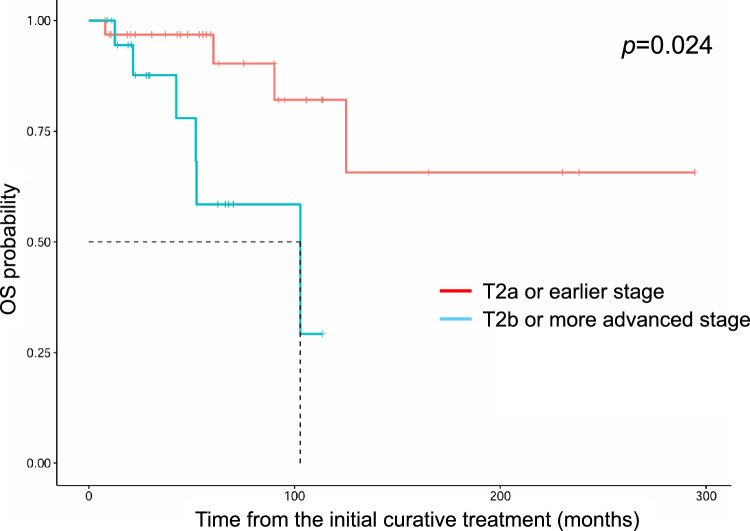
Kaplan-Meier curves for overall survival by T stage (≥T2b vs. ≤T2a). Patients with T2b or more advanced stage exhibited shorter overall survival than those with T2a or earlier stage. OS : Overall survival.

**Fig. 2.**
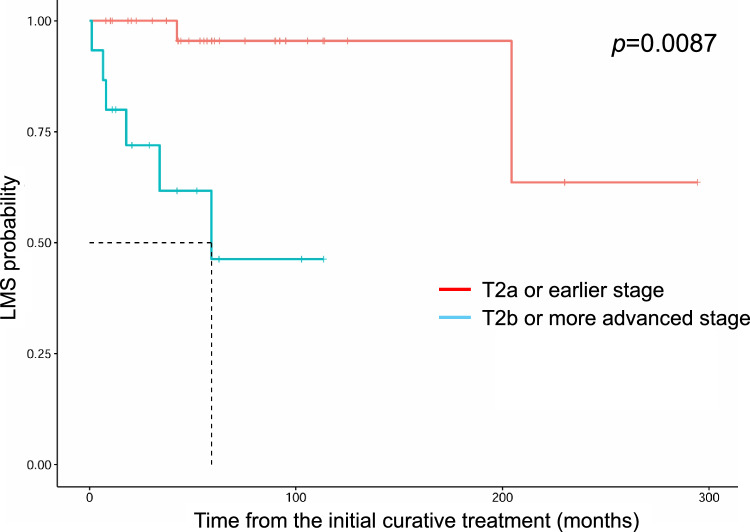
Kaplan-Meier curves for lymph node metastasis-free survival by T stage (≥T2b vs. ≤T2a). Patients with T2b or more advanced stage exhibited more lymph node metastases than those with T2a or earlier stage. LMS : lymph node metastasis-free survival.

The median follow-up time for the 52 patients was 50 months (7–294). Two- and five-year-adjusted survival rates were 97.4% and 93.7%, respectively. During follow-up, 10 patients (19%) died. Of these patients, three patients (6%) died of lung metastasis of eyelid SC (21, 52, and 92 months, respectively) (Table 2). The other seven patients died of other reasons including other cancers as well as noncancer disease. The log-rank test (Fig. 1) and multivariate analysis revealed that the patients with T2b or more advanced stages exhibited shorter overall survival compared with earlier stage patients (HR = 5.3, 95% CI = 1.2–23, *p* = 0.024) (Table 3). Local recurrence occurred in 15 patients (15/52, 29%) with a median interval of 24 months (3–243 months) **(**Table 4). Patients administered radiation therapy as the initial curative treatment recurred with high frequency (5/7, 71%), whereas patients treated surgically had a low rate of recurrence (10/45, 22%). Patients with negative surgical margins had an even lower recurrence rate (4/36, 11%), and multivariate analysis revealed that patients with negative surgical margins were less likely to recur locally (HR = 18, 95% CI 4.0–85, *p* = 0.00020). In Table 3, seven patients treated with radiation therapy were included as cases with positive surgical margins. To properly evaluate whether surgical margins were related to local recurrence, we added a multivariate analysis limited to 45 surgically treated patients. The multivariate analysis revealed that patients with positive surgical margins were more likely to recur locally (HR = 37, 95% CI = 5.9- 235, *p* = 0.00012). Pagetoid spread was not associated with recurrence (*p* = 0.91). Metastasis was observed in 14 patients (27%) during follow-up, including five patients who had metastasized at the time of the initial diagnosis (Table 5). The median interval of metastasis, except for these five patients, was 34 months (range 1–188 months). First metastasis site was the ipsilateral parotid gland or cervical lymph nodes in 13 patients and the lung directly in one patient. Among 13 patients with first metastasis to the ipsilateral parotid gland or cervical lymph nodes, 2 patients eventually metastasized to the lungs, 1 to the lung and cervical spine, and 1 to the lung and the liver. In five patients with distant metastasis, three patients died in 8, 11, and 25 months after the first distant metastasis. The remaining two patients are alive at 8 and 25 months after distant metastasis; their prognosis might be poor because there is no established chemotherapeutic drug regimen. Log-rank test (Fig. 2) and multivariate analysis revealed that T2b or advanced stage patients exhibited more lymph node metastases (HR = 82, 95% CI = 2.6–2.6e+03, *p* = 0.013) compared with T2a or earlier stage patients (Table 3). Pagetoid spread, as well as diffuse type, was not associated with recurrence, overall survival, lymph node metastasis-free survival, and distant metastasis-free survival.

**Table 2:** Details of 3 patients who died of sebaceous carcinoma.

Case	Age Sex	Location	TNM Classification	Initial curative treatment	Post-surgical treatment	Surgical margin	OS (months)	DMFS (months)	Duration from distant metastasis to death	Cause of death	Recurrence	Node metastasis
3	69 F	Left Lower Middle	T2aN0M0	RT (Electron)	NA	NA	90	64	26	Lung metastases	+	−
6	84 M	Left Upper Medial	T2bN0M0	Excision	−	Negative	52	41	11	Lung metastases	+	+
15	77 M	Left Both Medial	T4aN1M0	Exenteration + ND	IMRT 70Gy/35fr	Positive	21	12	9	Lung metastases	+	+

M, male; F, female; ND, neck dissection; RT, radiation therapy; IMRT, intensity modulated radiation therapy; NA, not available; OS, overall survival; DMFS, distant metastasis-free survival

**Table 3:** Univariate and multivariate Cox regression analyses for the prediction of overall survival, recurrence-free survival, lymph node metastasis-free survival and distant metastasis-free survival.

Overall survival	Univariable		Multivariable	
Factors	HR (95%CI)	*p*-value	HR (95%CI)	*p*-value
Location (medial vs. middle or lateral)	0.98 (0.28–3.5)	0.97	NA	NA
Site (lower vs. upper)	0.86 (0.21–3.5)	0.84	NA	NA
Site (Both vs. upper)	7.6 (0.66–88)	0.1	NA	NA
Type (diffuse vs. nodular)	3.7e–08 (0–Inf)	0.99	NA	NA
T stage (≥T2b vs. ≤T2a)	5.1 (1.2–21)	0.024*	5.3 (1.2–23)	0.024*
N stage (N1 vs. N0)	2.7 (0.31–23)	0.37	1.3 (0.12–14)	0.83
Stage at treatment (early vs. late)	2.2 (0.56–8.8)	0.26	2.1 (0.52–8.7)	0.3
Initial treatment(radiation vs. surgery)	0.38 (0.047–3.0)	0.36	0.34 (0.040–2.9)	0.32
Surgical margin	1.2 (0.34–4.3)	0.76	NA	NA
Pagetoid spread	2.6 (0.29–24)	0.39	2.0 (0.19–22)	0.56
Vessel invasion	2.3 (0.26–20)	0.45	NA	NA

Recurrence-free survival	Univariable		Multivariable	
Factors	HR (95%CI)	*p*-value	HR (95%CI)	*p*-value
Location (medial vs. middle or lateral)	1.6 (0.56–4.6)	0.38	1.1 (0.33–3.9)	0.85
Site (lower vs. upper)	1.8 (0.61–5.5)	0.28	NA	NA
Site (Both vs. upper)	3.2 (0.37–28)	0.29	NA	NA
Type (diffuse vs. nodular)	1.5 (0.19–12)	0.72	NA	NA
T stage (≥T2b vs. ≤T2a)	1.9 (0.64–5.8)	0.24	2.0 (0.49–8.2)	0.34
N stage (N1 vs. N0)	1.4 (0.18–11)	0.75	NA	NA
Stage at treatment (early vs. late)	1.0 (0.35–3.1)	0.95	NA	NA
Initial treatment (radiation vs. surgery)	2.6 (0.83–7.8)	0.1	0.30 (0.065–1.4)	0.12
Surgical margin	9.8 (2.7–35)	0.00051*	18 (4.0–85)	0.0002*
Pagetoid spread	0.89 (0.11–6.9)	0.91	NA	NA
Vessel invasion	3.1 (0.63–15)	0.16	0.85 (0.13–5.5)	0.86

Lymph node metastasis-free survival	Univariable		Multivariable	
Factors	HR (95%CI)	*p*-value	HR (95%CI)	*p*-value
Location (medial vs. middle or lateral)	1.5 (0.38–6.1)	0.56	NA	NA
Site (lower vs. upper)	1.1 (0.24–5.1)	0.9	9.0 (0.50–163)	0.14
Site (Both vs. upper)	12 (1.1–126)	0.039*	18 (0.86–375)	0.063
Type (diffuse vs. nodular)	2.8 (0.33–23)	0.35	NA	NA
T stage (≥T2b vs. ≤T2a)	17 (2.1–143)	0.0087*	82 (2.6–2.6e+03)	0.013*
N stage (N1 vs. N0)	NA	NA	NA	NA
Stage at treatment (early vs. late)	0.34 (0.067–1.7)	0.18	0.076 (0.0061–0.93)	0.044*
Initial treatment (radiation vs. surgery)	2.4 (0.51–11)	0.27	3.3 (0.32–34)	0.31
Surgical margin	1.1 (0.26–5.0)	0.86	NA	NA
Pagetoid spread	2.8 (0.31–24)	0.36	NA	NA
Vessel invasion	3.0 (0.35–25)	0.32	0.28 (0.013–6.2)	0.42

Distant metastasis-free survival	Univariable		Multivariable	
Factors	HR (95%CI)	*p*-value	HR (95%CI)	*p*-value
Location (medial vs. middle or lateral)	2.2 (0.37–13)	0.39	2.1 (0.25–17)	0.5
Site (lower vs. upper)	0.90 (0.081–9.9)	0.93	NA	NA
Site (Both vs. upper)	5.6e+09 (0–Inf)	0.99	NA	NA
Type (diffuse vs. nodular)	1.4e–08 (0–Inf)	0.99	NA	NA
T stage (≥T2b vs. ≤T2a)	9.2 (1.0–84)	0.048*	8.2 (0.81–84)	0.075
N stage (N1 vs. N0)	4.2 (0.46–39)	0.2	1.6 (0.12–21)	0.74
Stage at treatment (early vs. late)	0.78 (0.13–4.7)	0.79	NA	NA
Initial treatment (radiation vs. surgery)	0.99 (0.11–9.0)	0.99	NA	NA
Surgical margin	1.3 (0.21–7.6)	0.8	NA	NA
Pagetoid spread	4.5 (0.43–47)	0.21	4.1 (0.28–60)	0.3
Vessel invasion	3.7 (0.39–35)	0.26	0.74 (0.044–13)	0.83

HR, hazard ratio; CI, confidence interval; Inf, infinity; NA, not available; *, *p*-value < 0.05

**Table 4:** Details of 15 cases of local recurrence.

Case	Age Sex	Location	TNM classification	Initial curative treatment	Post-surgical treatment	Surgical margin	Period until recurrence (months)	Node metastasis	Distant metastasis
1	69 F	Left Lower Middle	T2aN0M0	RT (Electron)	NA	NA	50	+	−
2	58 M	Left Lower Lateral	T2aN0M0	RT (Electron)	NA	NA	18	−	−
3	65 M	Right Upper Lateral	T2aN0M0	RT (Electron)	NA	NA	139	−	+
4	74 F	Right Upper Medial	T3bN0M0	RT (Electron)	NA	NA	39	+	−
5	74 M	Right Upper Medial	T4aN0M0	RT (IMRT+Electron)	NA	NA	6	+	−
6	84 M	Left Upper Medial	T2bN0M0	Excision	−	Negative	37	+	+
7	50 F	Right Lower Medial	T1aN0M0	Excision	−	Negative	243	−	−
8	90 F	Right Upper Middle	T1aN0M0	Excision	RT (interruption due to heart failure)	Positive	17	−	−
9	64 M	Left Lower Middle	T2aN0M0	Excision + reconstruction	−	Negative	24	+	−
10	81 F	Left Upper Middle	T2aN0M0	Excision	MMC eye drops	Positive	43	−	−
11	62 F	Left Upper Medial	T2aN0M0	Excision + reconstruction	−	Positive	42	−	−
12	56 F	Right Lower Medial	T2aN0M0	Excision	−	Positive	3	−	−
13	80 F	Right Lower Medial	T3cN0M0	Excision + reconstruction	−	Positive	14	−	−
14	74 F	Left Lower Lateral	T4aN0M0	Excision + reconstruction	−	Negative	15	+	−
15	77 M	Left Both Medial	T4aN1M0	Exenteration + ND	IMRT 70Gy/35fr	Positive	13	N1	+

M, male; F, female; RT, radiation therapy; ND, neck dissection; NA, not available; MMC, mitomycin C; IMRT, intensity modulated radiation therapy; Table 4 is in order of the initial curative treatment.

**Table 5:** Details of 14 metastasized patients.

Case	Age Sex	Location	TNM Classification	Initial curative therapy	Post-surgical therapy	Surgical margin	First location of metastasis	Neck dissection	Radiation to neck	Other location of metastasis	NMFS (months)	DMFS (months)	OS (months)
Node metastasis only													
2	58 M	Left Lower Lateral	T2aN0M0	RT (Electron)	NA	NA	C	+	+	Axillary, contralate-ral C	188	238 (No meta)	238 (alive)
9	64 M	Left Lower Middle	T2aN0M0	Excision + reconst- ruction	−	Negative	P	+	−	−	43	165 (No meta)	165 (alive)
4	74 F	Right Upper Medial	T3bN0M0	RT (Electron)	NA	NA	P	+	−	−	59	66 (No meta)	66 (alive)
5	74 M	Right Upper Medial	T4aN0M0	RT (IMRT + Electron)	NA	NA	P+C	+	+	−	6	29 (No meta)	29 (alive)
14	74 F	Left Lower Lateral	T4aN0M0	Excision + reconstruction	−	Negative	P	+	−	−	8	27 (No meta)	27 (alive)
18	81 F	Right Upper Medial	T2bN1M0	Excision + ND	RT	Positive	C	+	+	−	0(N1)	22 (No meta)	22 (alive)
19	85 F	Right Upper Medial	T3aN1M0	Excision + ND	−	Negative	C	+	−	−	0(N1)	8 (No meta)	8 (alive)
20	59 F	Right Upper Middle	T3cN1M0	RT (Interstitial brachytherapy)	NA	NA	C	+	+	P	0(N1)	70 (No meta)	70 (alive)
21	79 F	Right Upper Medial	T4aN1M0	Exenteration + ND	−	Negative	P	+	+	−	0(N1)	13 (No meta)	13 (alive)
Node metastasis + Distant metastasis													
6	84 M	Left Upper Medial	T2bN0M0	Excision	−	Negative	P+C	+	−	Lung, cervical vertebrae	34	41	52 (dead)
16	39 M	Right Upper Middle	T2bN0M0	Excision + reconstruction	−	Negative	C	+	+	Lung	18	41	67 (alive)
17	75 M	Left Both Medial	T4aN0M0	Exenteration	−	Negative	C	+	−	Lung Liver	1	13	19 (alive)
15	77 M	Left Both Medial	T4aN1M0	Exenteration + ND	IMRT	Positive	P	+	+	Lung	0(N1)	12	21 (dead)
Distant metastasis only													
1	69 F	Left Lower Middle	T2aN0M0	RT (Electron)	NA	NA	Lung	NA	NA	−	90 (No meta)	64	90 (dead)

M, male; F, female; RT, radiation therapy; ND, neck dissection ; IMRT, intensity modulated radiation therapy; P, parotid gland; C, cervical lymph node; NA, not available; NMFS, node metastasis-free survival; DMFS, distant metastasis-free survival; OS, overall survival; Table 5 is in order of T stage and N stage in each metastasis site.

## Discussion

We examined the clinical course of rare eyelid SC in Japanese. Two- and five-year adjusted survival rates were 97.4% and 93.7%, respectively. In a series of 100 patients, Sa et al. reported a 93.8% two-year survival rate and a 92.0% five-year survival rate with a median follow-up period of 31.5 months [14]. Esmaeli et al. reported a 93.3% two-year survival rate and a 79.3% five-year survival rate in 50 cases of eyelid SC [15]. Although differences in sample size, follow-up time, racial differences, and variability in management must be considered, the survival rate of our cohort was relatively good.

In our cohort, local recurrence was observed in 29% (15/52), lymph node metastasis in 25% (13/52), distant metastasis in 8% (4/52), and tumor specific mortality was 6% (3/52). According to multiple case reports of over 50 cases published since 2000, local recurrence occurred in 6-44% [14, 16–25], lymph node metastasis in 3-23% [15, 16, 19–25], distant metastasis in 0-14% [14–16, 19–25], and tumor specific mortality was 2-13% [14–26]. Although lymph node metastasis of our cohort was slightly higher, the major reason for this is that our cohort included recurrent patients who had first been treated at other hospitals. For the 39 cases initially treated at our hospital, lymph node metastasis occurred in 18% (7/39) and it was on par with other reports.

In other Japanese cohorts, 3% (4 patients in a series of 116 patients, follow-up period of at least 24 months) [18] and 2% (one patient in a series of 63 patients, at a median follow-up period of 4.2 years) died of eyelid SC during the follow-up period [22]; whereas in our cohort, three patients (6%) died of eyelid SC. Mortality rates in Japanese cohorts are slightly lower than in other countries (4–13%) [14–17, 19, 21, 23–26]. Tumor presentation patterns, such as nodular and diffuse, are different between Western and Asian countries [21]. Caucasians have a higher prevalence of diffuse eyelid thickening, and Asian patients tend to have nodular presentation. A previous study reports that patients with local recurrence and metastasis exhibited a different tumor presentation pattern in patients without local recurrence and metastasis (*p* = 0.006) [27]. The difference in tumor presentation patterns may affect the outcome differences between Western and Asian countries. However, our cohort found no significant difference in local recurrence, metastasis, and overall survival between nodular and diffuse tumor presentation patterns. There were only four patients of diffuse type in our cohort, therefore, it would be better to examine differences in tumor presentation patterns in a larger number of cases.

Previous studies indicate that larger tumor size and more advanced T categories are associated with worse overall survival [14, 17, 21, 26]. Similarly, in our study, T stage (T2a or earlier vs. T2b or more advanced) was a predictor of overall survival. With respect to pagetoid spread, Zhou et al. found that patients with pagetoid spread were significantly more likely to have cumulative tumor-related mortality [26]. In our study, however, pagetoid spread was not a predictor of overall survival as well as other studies [21, 28].

In our analysis, positive surgical margins increased local recurrence. We did not perform Mohs micrographic surgery, although we did perform local resection with a 2-5 mm surgical margin. Resection was difficult when the tumor was large and close to the canthus, because the eyelid is an important structure that maintains one’s facial appearance and is close to the lacrimal gland, lacrimal sac, and lacrimal drainage system. However, because negative surgical margins are associated with reduced local recurrence, tumors should be resected with negative margins.

Xu et al. reported that diagnostic delay, orbital involvement, Ki67 and initial surgery of Mohs micrographic surgery were independent influencing factors for recurrence [16]. Among these factors, we considered the time from the symptoms’ onset to the initial curative treatment. In our study, treatment delay did not increase local recurrence but it did increase lymph node metastasis.

Even if eyelid SC is controlled locally, metastases to regional lymph nodes and even distant organs are difficult to treat. In our practice, head and neck surgeons perform superficial parotidectomy with neck dissection for metastases to regional lymph nodes. If extranodal extension out of lymph node structure or four or more lymph node metastases are histopathologically observed, postoperative radiation therapy is administered. Distant metastasis was prevented in 69% with lymph node metastasis (9/13, median follow-up period: 22 months, range 7-122) and the treatment seemed effective. Zhou et al. [26] and Sa et al. [14] reported that N1 is a risk factor of death from disease, although, in our cohort N1 was not associated with overall survival. For metastases to distant organs, customized chemotherapy is administered because there is no standard evidence-based chemotherapeutic drug regimen. However, treatment outcomes are not good, and efforts must be made to prevent metastasis to distant organs. The development of effective chemotherapy and immunotherapy based on eyelid SC molecular biology is needed. There are reports that predictors of lymph node and distant metastasis include medial or lateral canthus involvement, perivascular invasion, tumor diameter >10 mm [17], and T stage [14]. TNM stage, orbital invasion, orbital exenteration [19], tumor size [18], tumor diameter >20mm and positive surgical margin [21] are also reported as predictors of lymph node metastasis. In our study, T stage (T2b or more advanced stage vs. T2a or earlier stage) was the strongest predictor of lymph node metastasis. Taken together, these reports suggest that larger and invasive tumors pose a greater risk of metastasis.

In conclusion, 52 Japanese patients with eyelid SC were analyzed. Some of our findings are similar to those of other groups, whereas some are different in part, mainly because SC is a rare tumor with a small sample size, follow-up period, racial differences, variability in management that may affect the results. In our study, a T stage worse than T2b was associated with worse overall survival as well as lymph node metastasis compared with T2a or better. Therefore, eyelid SC must be diagnosed accurately and treated while the tumor is small. In addition, surgery should be performed with a negative margin as a positive margin is a risk for local recurrence.
